# Incorporating Research and Evaluation into Pandemic Influenza Vaccination Preparedness and Response

**DOI:** 10.3201/eid2004.140224

**Published:** 2014-04

**Authors:** Tom T. Shimabukuro, Stephen C. Redd

**Affiliations:** Centers for Disease Control and Prevention, Atlanta, Georgia, USA

**Keywords:** Pandemic influenza, emergency preparedness, vaccination, evaluation, research, influenza, viruses

This issue of Emerging Infectious Diseases contains 2 articles that address critical elements of implementing large-scale local-level vaccination programs in response to a public health emergency. In addition to describing activities undertaken during the 2009 pandemic of influenza A(H1N1) virus (pH1N1) and subsequent program evaluation and lessons learned, the articles highlight the critical role for scientific evaluation in improving our ability to respond to emergencies (*1*,*2*).

Saha et al. report on assessment of the efficiency of public health–managed large-scale vaccination clinics, referred to as points of dispensing (PODs), to administer pH1N1 vaccine in densely populated Los Angeles County, California, USA (*3*). The authors examined rates of visits to PODs according to patients’ socioethnic characteristics and assessed factors affecting vaccination throughput (doses administered per hour). Their evaluation provides information about optimal placement of PODs in the community and possible strategies to improve their operational efficiency.

Marcello et al. describe the experience of New York City, New York, USA, in using its Citywide Immunization Registry to capture information about pH1N1 vaccine doses administered during the response (*4*). Immunization information systems (IISs) are commonly used to document administration of recommended childhood vaccinations; however, routine adult participation has historically been low (*5*). During New York City’s pH1N1 vaccination program, the health department required all providers to register with the Citywide Immunization Registry and report doses administered. The New York City experience demonstrates the feasibility and potential usefulness of expanding mandatory IIS reporting to all types of providers during a pandemic influenza vaccination program as a means of monitoring progress and managing supply and distribution. The article also reveals limitations of IISs as they existed in 2009 and 2010.

Los Angeles County and New York City were able to conduct meaningful program evaluation because public health officials had the foresight to incorporate evaluation into emergency planning and response and commit valuable time and resources to conduct health services research during the height of pH1N1 vaccination. Los Angeles County health officials coordinated a meticulous data collection effort from 101 POD events held during a 6-week period from October through December 2009. In New York City, a substantial outreach and education program was necessary to incorporate providers of vaccines to adults and others not accustomed to IIS reporting into the program to acquire the most comprehensive and timely information possible about vaccine doses administered.

For health departments, the decision to commit to planning for and conducting research and evaluation during a public health emergency is complicated by the competing priority of providing direct services to persons and populations in need. In addition to balancing the effort needed to plan and conduct the public health response and the research or evaluation effort, other uncertainties impose limitations on research efforts during emergencies. In the case of research conducted during the 2009 influenza A(H1N1) pandemic, the inherent difficulty of projecting demand for vaccination, combined with delays and uncertainty around the timing of availability of pH1N1 vaccine, were serious challenges for the vaccination program and for its evaluation (*6*). 

Although researchers have to make assumptions about the event under study, a high degree of flexibility is necessary. Public health emergencies often present unforeseen circumstances and influenza pandemics are among the least predictable of all emergencies. Public health priorities during a pandemic response can change quickly on the basis of disease characteristics, resource constraints, and the potential for social disruption. Planning evaluation efforts for different pandemic scenarios and being nimble enough to rapidly adapt to shifting priorities are essential qualities for any research and evaluation program. Research planners need to be able to identify and address key response questions under conditions of much less certainty than in other research efforts.

Despite these challenges, invaluable knowledge is gained from well-planned and well-conducted (and appropriately resourced) health services research during an event. Tabletop and functional exercises are useful tools for organizations to expand knowledge, assess readiness, and identify deficiencies (*7*,*8*). Yet they rarely approach the intensity, complexity, and duration of a real event. Data obtained during a response to an actual public health emergency provide the best (and perhaps only) source of information for program evaluation under conditions in which the public health system is severely stressed. To address knowledge gaps in preparedness, public health authorities must strike an appropriate balance between conducting research, evaluating program efforts, and providing services during a public health emergency (*2*). Publishing the results of such evaluations is also essential to permit others who are planning vaccination campaigns during emergencies to benefit from the experiences in Los Angeles County and New York City.

Human infection with influenza A(H3N2) variant virus (*9*) and avian influenza A(H7N9) virus (*10*) and continued sporadic cases of infection with highly pathogenic avian influenza A(H5N1) virus (*11*)—all viruses with pandemic potential—remind us that we must remain vigilant in our preparedness. We encourage health officials and leaders of health care organizations at all levels to identify the critical questions that will affect future emergencies and design research efforts into emergency preparedness planning to take advantage of these rare opportunities to learn and improve the nation’s response capability. For vaccination programs during influenza pandemics, priorities for evaluation include the following: efforts to improve situational awareness; efforts to identify and vaccinate populations prioritized for vaccination, including vulnerable populations and groups prioritized because of occupation; strategies to balance vaccine allocation to the existing, largely private, vaccination system, with large-scale vaccination venues (i.e., PODs); and strategies to build systems for pandemic influenza response that also improve seasonal influenza vaccination programs.

Although we recognize that smaller and less well-resourced organizations may be challenged in their ability to conduct large-scale sophisticated evaluation, we believe that learning by doing is possible for any organization, provided leaders and planners are willing to make the commitment. Even modest evaluation efforts will increase knowledge and advance preparedness.
